# Advanced Biomedical Applications of Reactive Oxygen Species-Based Nanomaterials in Lung Cancer

**DOI:** 10.3389/fchem.2021.649772

**Published:** 2021-04-07

**Authors:** Nan Zhao, Hua Xin, Lening Zhang

**Affiliations:** Department of Thoracic Surgery, China-Japan Union Hospital of Jilin University, Changchun City, China

**Keywords:** reactive oxygen species1, nanomaterials2, lung cancer3, anti-cancer4, A549 cells5, apoptosis6

## Abstract

Over the years, lung cancer remains the leading cause of cancer deaths in worldwide. In view of this, increasingly importance has been attached to the further optimization and improvement of its treatment. Reactive oxygen species (ROS) play a key role in regulating tumor development and anti-cancer treatment. Recently, the development of nanomaterials provides new platforms for ROS-based cancer treatment methods, which can help to reduce side effects and enhance anti-cancer effects. In recent years, a variety of lung cancer treatment models have been reported, such as chemodynamic therapy (CDT), photodynamic therapy (PDT), radiation therapy (RT) and controlled drug release (CDR). In this review, we are going to discuss the possible mechanism of action and current research status of ROS-based nanomaterials in the treatment of lung cancer in order to provide constructive ideas for relative research and expect this work could inspire the future development of novel lung cancer treatments.

## Introduction

Reactive oxygen species (ROS) is a collective term used to describe chemicals which were formed from incomplete reduction of oxygen (D'Autreaux and Toledano, 2007). It mainly consists of superoxide anion (O_2_^−^), hydrogen peroxide (H_2_O_2_), hydroxyl radical (OH) and singlet oxygen (1O_2_), etc. (Auten and Davis, 2009; Nosaka and Nosaka, 2017) ROS are believed to be necessary to regulate the following normal physiological functions: 1) cell cycle progression and proliferation; 2) differentiation; 3) migration; 4) cell death. On the one hand, endogenous ROS are mainly produced in mitochondria, which plays an important physiological role in cell signaling and metabolism. On the other hand, ROS also participate in the regulation of many biological processes. Cancer cells have higher ROS levels than normal cells due to the increased metabolism and mitochondrial dysfunction. The increase of ROS production in cancer cells is a biochemical and molecular change, which is necessary for: 1) tumorigenesis; 2) progression; 3) metastasis; 4) tumor resistance to chemotherapy(Tafani et al., 2016). Therefore, the increase of ROS in cancer cells can provide an opportunity to activate various ROS-induced cell death pathways or inhibit the resistance of cancer cells to chemotherapy. This can be achieved by using increased ROS generation, inhibition of antioxidant defenses or even a combination of the two (Galadari et al., 2017). Excessive intracellular ROS levels can cause damage to proteins, nucleic acids, lipids, membranes and organelles, thus activating cell death processes such as apoptosis. What’s more, ROS can effectively regulate the cell signal transduction and major pathways of apoptosis mediated by: 1) mitochondria; 2) death receptors; 3) endoplasmic reticulum (Yang et al., 2019). Given all that, ROS-based cancer therapies will open up possibilities to a new generation of cancer treatment strategies.

All over the world, there were an estimated 9.6 million cancer deaths and 18.1 million new cancer cases in 2018. Lung cancer remains the leading cause of cancer incidence and mortality, with 2.1 million new cases and 1.8 million deaths. In both males and females, lung cancer is the most commonly diagnosed cancer (11.6%) and the leading cause of cancer death (18.4%) (Bray et al., 2018; Siegel et al., 2020). Among all the treatments of lung cancer, surgery, chemotherapy, radiotherapy, targeted therapy and other surgeries are the main ways to treat non-small cell lung cancer. Unfortunately, many lung cancer patients are already found to be in the advanced stage when seeking medical help. Considering the difficulty to carry out effective surgical treatment for advanced lung cancer, so comprehensive systemic anti-tumor therapy comes to be a necessary way to prolong the survival time. As an important part of lung cancer treatment, non-surgical treatment can not only target the primary lesion but also the metastatic lesion. Hence it is imperative to optimize and improve non-surgical treatment in lung cancer.

Aiming to improve the reflection efficiency and biological interaction, nanotechnology has brought a new round of technological revolution to ROS science. This promotes the emergence of many multifunctional nanomaterials, including ROS generation, conversion and consumption. Doing this will achieve better therapeutic effects. As materials grow in size to the nanoscale, they exhibit new and unique properties (Auffan et al., 2009). In the treatment of cancer, the small size of nanomaterials contributes to the penetration of the nanosystem into tissues, enrichment of tumor tissues, cellular uptake, intracellular transport and elimination of the nanosystem(Yang et al., 2018). ROS nanotechnology intersects chemistry and biology at the nanoscale to construct nano-drugs with ROS regulation function and improve therapeutic effect, providing new directions for tumor therapy.

## Mechanism of ROS-Based Lung Cancer Therapy

**FIGURE 1:**
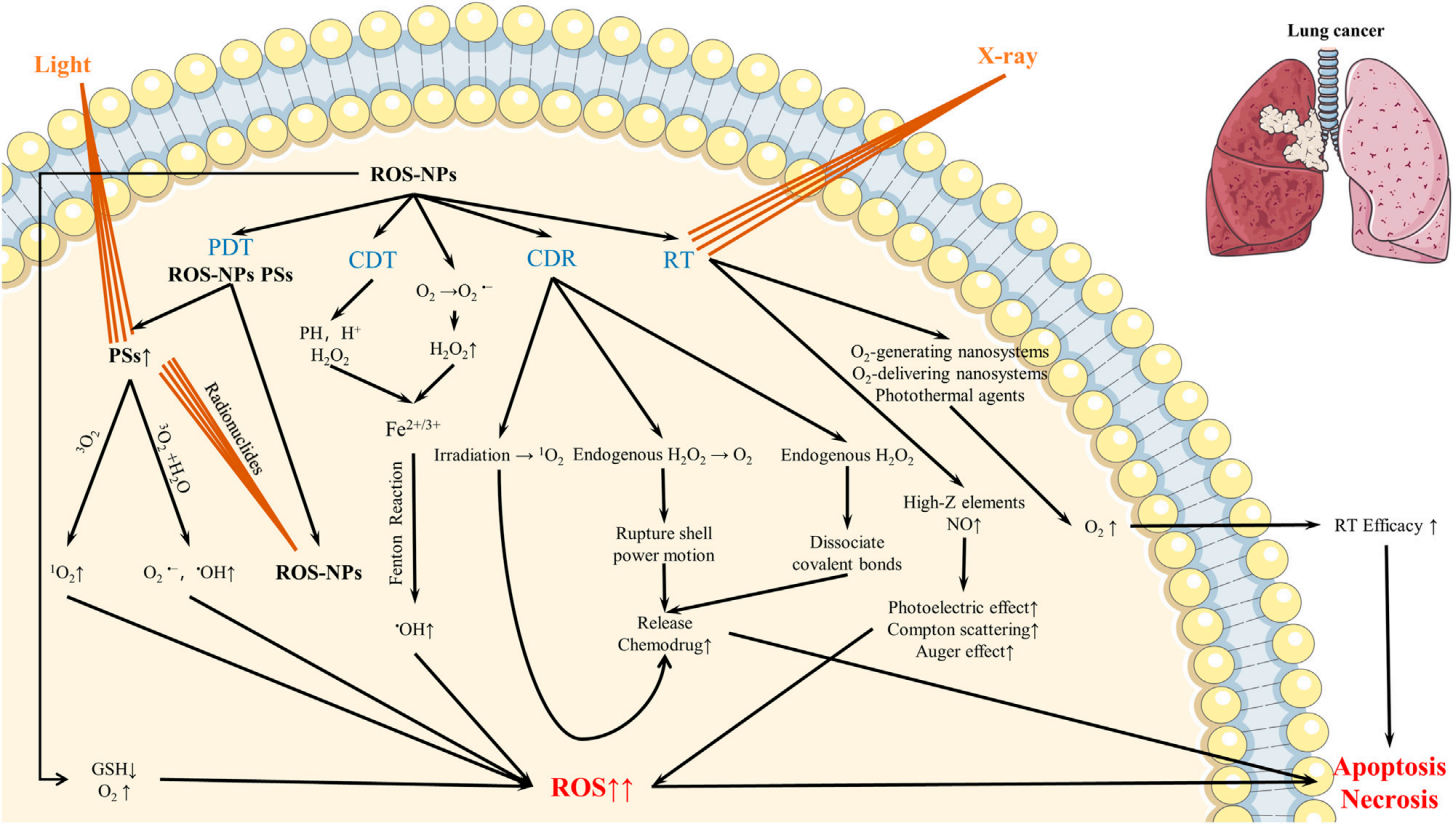
Mechanism of ROS-based nanomaterials in lung cancer therapy.

ROS play a key role in regulating tumor development and anti-cancer treatment. High levels of ROS are usually harmful to cells. When cells change from normal state to carcinomatous condition, ROS levels are gradually increased. Through the antioxidant defense system, ROS levels are maintained to a new stable equilibrium state, and ROS levels are regulated below the toxicity threshold to avoid oxidative damage. (Gorrini et al., 2013). Thus, tumor cells can be induced to die by the breakdown of antioxidant system caused by the accumulation of excessive ROS, which can be used in the treatment of cancer. Cancer cells rely on the endogenous antioxidant defense system to maintain the new redox homeostasis, while exogenous ROS intervention can disrupt this balance, causing the rise of ROS and thereby destroying cancer cells (Yang et al., 2019). Based on this, ROS-based nanomaterials can be applied to various treatment modes of lung cancer, such as CDT, PDT, RT and CDR (Figure 1). In the following sections, we introduce the advances in the application of these ROS-based nanomaterials in lung cancer treatment in recent years.

### Chemodynamic Therapy (CDT)

The unique biochemical characteristics of tumor microenvironment (TME), such as high GSH concentration, elevated H_2_O_2_ level and mild acidity make it possible to distinguish tumor tissue from normal tissue (Tang et al., 2019; Yang et al., 2019). We can use these intrinsic biochemical properties as an endogenous stimuli to target the activation of nanomaterials in tumor tissues and transform the chemicals in the tumor into ROS to cause tumor cell damage. Most nanosystems are designed to respond to intratumoral H^+^ and H_2_O_2_, thereby promoting the production of Fenton or Fenton-like reactions in TME, and facilitating the production of highly toxic OH to damage the cancer cells (Liu et al., 2019). The TME-activated localized Fenton reaction for cancer therapy mentioned above was termed as CDT by Zhang et al. in their 2016 report (Zhang et al., 2016). Lee et al. reported that SnFe_2_O_4_ nanocrystals could effectively transform endogenous H_2_O_2_ into highly active hydroxyl radicals through catalyzing heterogeneous Fenton reaction, causing apoptosis and death of lung cancer cells. Moreover, they proved and demonstrated that the key role to achieve selective elimination of cancer cells is the catalase for the first time (Lee et al., 2017). Das et al. showed synthesis iron oxide nanoparticles (IONPs) can induce apoptosis of lung cancer cells. Fe^2+^ ions can react with H_2_O_2_ through Fenton reaction to produce hydroxyl radical to destroy DNA. This research study may provide valuable information about therapeutic dose and side-effects in chemotherapy for drug delivery (Das et al., 2020).

The application of ROS-based nanomaterials to lung cancer CDT is a promising field, but there are few reports on it. By stimulating the endogenous biochemical characteristics of tumor tissues, tumor cytotoxic ROS can be generated without damaging normal tissues. CDT will be a new anti-cancer treatment with good efficacy and few side effects.

### Photodynamic Therapy (PDT)

PDT is composed of photosensitizer (PS), light and tissue oxygen. The corresponding wavelength irradiation can activate the PS, resulting in excessive intracellular ROS production, leading to apoptosis, death of cancer cells and stimulating the host immune system (Dolmans et al., 2003; Agostinis et al., 2011). Less toxic to normal tissues, PDT has organ function-sparing effects and lacks drug resistance mechanism. Thus, it can prolong the survival time and improve the quality of life of cancer patients who cannot be operated on. However, the ability of excitations light to penetrate tissue limits the clinical application of PDT (Fan et al., 2016). ROS-based nanomaterials promise to break through this barrier.

As a PS drug delivery system, nanoparticles (NPs) can increase the targeting and stability of PSs, reduce side effects, and protect the PSs to reach the cancer tissue successfully. Nanomaterials wrapped by cell membrane or protein coat can improve the defect of photosensitizers such as: 1) poor water solubility; 2) rapid blood clearance; 3) lack of effective targeting. Therefore, the wrapped nanomaterials have affinity with tumor blood vessels and tumor cell membranes to promote the uptake of PSs by tumor cells. Feng et al. synthesized a drug delivery system (Ng/Ce6@SCV) composed of hydrophobic PS, chlorin e6 (Ce6), gelatin nanogels (Ng) and coated stem cell membrane vesicles (SCV) as the outer shells. Ng/Ce6@SCV can slow the release of Ce6 to maintain high Ce6 concentration in the cancer tissues so that it can achieve more eminent anti-tumor efficacy (Feng et al., 2020) Nag et al. synthesized a nanohybrid (AuNP-PpIX) of protein assembled gold nanoparticles (AuNPs), attaching Protoporphyrin IX (PpIX) to the protein coat of the NPs. The protein coat gives the AuNP-PpIX a higher stability to show excellent anti-cancer efficiency upon irradiation on A549 cells through intracellular ROS generation (Nag et al., 2020). Zhou et al. synthesized the Nanoparticle of a Ru(II)-based photosensitizer and complementary Pt(II)-based building blocks, which was encapsulated into an amphiphilic polymer to encourage cell uptake and localization. The Nanoparticle can destroy cancer cells though the generation of ROS in the lysosomes under 2-photon near-infrared light (NIR) irradiation (Zhou et al., 2019). Tokarska et al. presented that they constructed a well-defined multilayer oil core nano capsules with tumor targeting by layer-by-layer assembly strategy. The multilayer nano capsules loaded with tetraphenyl porphyrin (TPP) have selective photodynamic activity in A549 cells, which can increase the uptake of TPP by cells, and generate ROS to enhance toxicity in A549 cells(Tokarska et al., 2019).

Nanomaterials can enhance the anti-cancer ability of PDT by stimulating the ROS generation in tumor cells. Sakr et al. showed a preparation of core-shell-shell magnetite-silica-titania nanoparticles (Fe_3_O_4_@SiO_2_@TiO_2_ NPs), stained with a polypyridyl ruthenium dye. Under 532 nm light illumination, the excellent ROS production is observed while the singlet oxygen generation is negligible. The advantages of the dyed NPs might be a significant increase in ROS production and it’s efficient action against low-oxygen cancerous cells (Sakr et al., 2016). NPs can destroy the endogenous antioxidant defense system by consuming intracellular antioxidant substances. Further, ROS improve to a higher level on the basis of PSs production of ROS and induce cell apoptosis to achieve the purpose of anti-cancer treatment. Sun et al. synthesized a kind of nanoparticle containing β-seleno diesters and porphyrin derivates. They introduced the selenoxide elimination reaction into nanomedicine mediated cancer therapy for the first time. The acrylic ester produced by selenoxide elimination reaction can consume intracellular GSH. Therefore, the intracellular ROS level of lung cancer cells is continuously increased, leading to cell apoptosis. Moreover, this combination of structural design and PSs would achieve a persistent production of ROS, which can be used for sustainable phototherapy even in dark conditions (Sun et al., 2019).

PDT, as one of the key means of ROS-based nanomaterials applied in the treatment of lung cancer, stands a good chance to embrace a broader clinical application prospect with the development of nano-platform technology.

### Radiation Therapy (RT)

RT is still one of the main clinical treatments for cancer, which uses ionizing radiation to cause physical and chemical changes, leading to DNA damage in cancer cells and then inhibiting tumor development. In clinical treatment, RT is used in local metastases of advanced lung cancer and to prolong the life cycle of patients. However, it often leads to collateral damage of normal tissues. ROS-based nanomaterials can improve the effect of RT through radiation sensitization of tumors, regulation of TME and improvement of radiation protection (Yang et al., 2019).

Ma et al. synthesized FePt NPs and assembled them on the surface of graphene oxide (GO) into novel FePt/GO nanosheets (FePt/GO NSs). The NSs can increase ROS production, improve the radiation sensitivity of lung cancer cells and induce cell apoptosis combined with the X-ray (Ma et al., 2019). Nanomaterials contained with high atomic number (high-Z) elements can release electrons (auger electrons and photoelectrons) and show strong photoelectric absorption ability for subsequent ROS generation. They have strong X-ray attenuation ability and can be used as radiation sensitizers. Li et al. prepared porous platinum nanoparticles (PtNPs) to effectively enhance RT in lung cancer cells, taking into account the combined benefits of photoelectric absorption ability and oxygen generation capability in the high-Z elements. PtNPs can significantly deposit X-ray radiation energy and covert endogenic H_2_O_2_ to O_2_ within the lung cancer cells in order to increase ROS stress, DNA damage and cell cycle arrest(Li et al., 2019). The application of ROS-based NPs to RT will improve the therapeutic effect of advanced lung cancer, which deserves further study and discussion.

### Controlled Drug Release (CDR)

Chemotherapy is one of the main therapeutic methods for lung cancer, but the resistance of chemotherapy drugs and the damage to normal tissues lead to unsatisfactory therapeutic effect(Thakor and Gambhir, 2013; He and Shi, 2014). In recent years, ROS - mediated CDR can initiate endogenous drug release in the tumor region and has been extensively developed (Tapeinos and Pandit, 2016). At present, ROS-mediated CDR can be divided into three categories: 1) endogenous H_2_O_2_ directly activated CDR; 2) endogenous H_2_O_2_ breakdown and O_2_ generation activated CDR; 3) exogenous physical irradiation produce ^1^O_2_ activated CDR (Yang et al., 2019).

ROS-based nanomaterials activate specific chemical reactions by ROS generation, causing targeted release of anti-cancer drugs, reducing damage to normal tissues, increasing drug concentration in tumor tissues and improving the therapeutic effect of anti-cancer drugs. A PH/ROS-responsive micelle drug delivery system was developed by Zhang et al. Doxorubicin (DOX) and α-tocopheryl succinate (TOS) were released in an environment with rich in ROS and acid of lung cancer cells. TOS further induced ROS production, accelerated DOX release, and induced apoptosis of lung cancer cells (Zhang et al., 2020). Seah et al. reported a photodynamic method for triggering drug release from NPs via ROS - mediated polymer degradation under NIR irradiation. Paclitaxel and 2, 3-naphthalene phthalocyanine bis (trihexylsiloxane) were co-encapsulated in biotin-decorated poly (ethylene glycol) polythioketal micelles bundles as anti-cancer drugs and photosensitizers, respectively. NIR activates naphthalocyanine to produce ROS, cleaved the thioketone groups in the micelle, release the encapsulated paclitaxel, and increase the cell uptake *in vitro* to achieve better anti-cancer therapeutic effect (Seah et al., 2018).

ROS-mediated CDR provides a new way for nano-drugs to accurately release anti-cancer drugs in lung cancer tissues. This treatment model provides a new way of thinking for optimizing anti-cancer drugs and improving the curative effect of chemotherapy, which will be a crucial instrument in clinical anti-cancer treatment.

### Synergistic Therapy

Single treatment modes often fail to achieve ideal cancer treatment expectations due to the diversity and complexity of tumors. Recurrence, drug resistance and mutation of tumor tissues often lead to unsatisfactory anti-cancer treatment effect (Chen et al., 2014). Therefore, it is imperative to shift from single-mode treatment to multi-mode cooperative treatment, and integrate the advantages of multi-mode treatment to make up for the disadvantages, so as to improve the anti-cancer treatment effect (Fan et al., 2017; Sun et al., 2017). The ROS-based nanomaterials developed on the basis of ROS science have been applied to a variety of anti-cancer treatment strategies (CDT, PDT, RT, and CDR). Thus, we hope that multi-mode therapy can be activated synchronously or successively through a specific stimulus and different therapeutic functional modules of ROS-based nanomaterials can be activated through multiple specific stimuli, so as to achieve the purpose of collaborative therapy. The development of multi-functional ROS-based nanomaterials makes multi-mode collaborative treatment possible.

Guo et al. reported a new type of platinum (IV) complex-based polyprodrug for first time, which can produce high level of ROS under light irradiation and *in situ* to produce highly toxic platinum (II) as a chemotherapeutic drug in a PDT-like process without the use of PS or consumption of oxygen. The nanogels can continuously release drug and reverse drug resistance for combined chemotherapy-photodynamic therapy (Guo et al., 2018). Cai et al. prepared a chlorin-lipid nanovesicle using Ce6 and phospholipids as conjugations. ^131^I-labeled bovine serum albumin (^131^I-BSA) was loaded into nanocapsules as an internal light source, and Ce6 was stimulated to produce ROS for the treatment of lung cancer combined with RT and PDT (Cai et al., 2020). Hauser et al. used iron oxide NPs, combining with cell penetrating peptide TAT, and radiation to synergistically improve anti-cancer ability. Radiation irradiates lung cancer cells to produce H_2_O_2_ and iron oxide NPs catalyze Fenton reaction, significantly increasing the production of radiation-related ROS. Combined with radiotherapy, synergistic combination therapy can be produced in the lung cancer (Hauser et al., 2016; Sun et al., 2018). Yue et al. synthesized a ROS-responsive drug with the PS Ce6, TL-CPT (camptothecin conjugated with thioketal linker) and carboxyl-mPEG were loaded on the c nanoparticles (UCNPs) which were named Ce6-CPT-UCNPs. Ce6 generates ROS triggering thioether linker oxidation under NIR irradiation and promotes the release of the anti-cancer drug into the cytoplasm of lung cancer cells. The Ce6-CPT-UCNPs were successfully used for fluorescence imaging, and simultaneous combined chemotherapy and PT of lung cancer under 980 nm laser irradiation. This should be first report (Yue et al., 2016).

Synergistic treatment combines the advantages of various treatment methods and acts synergistically in anti-cancer treatment to achieve therapeutic effects that are difficult to be achieved by single treatment. By interweaving treatments with different mechanisms of action, more effective antitumor manifestations can be achieved. Therefore, in-depth study on anti-cancer mechanism of ROS-based nanomaterials is the premise of multi-mode collaborative treatment, which still needs further researching.

## Conclusion and Outlook

In conclusion, the treatment of lung cancer with ROS-based nanomaterials has been extensively studied, but many projects are still in the exploratory stage and need further improvement. We reviewed the application of ROS-based nanomaterials in lung cancer treatment in recent years from CDT, PDT, RT and CDR. Many studies have demonstrated the potential application of ROS-based nanomaterials in the field of lung cancer treatment, but these studies cannot be immediately pressed into service to benefit patients. We hope that the synthesis of multifunctional nanomaterials through some ingenious designs will have a synergistic effect on the treatment of lung cancer, while optimize the synthesis process, quality, cost and production. Through further research on ROS-based nanomaterials, the future research should focus on materials can be synthesized to achieve precision, safety and individualized therapeutic effects. Most of the current research projects are still limited to the cell and animal level. Hence, we have to take into account the complexity of human body, the applicability of clinical treatment and the application from “bench to bedside.” With the development of nanotechnology and biological sciences, we can be optimistic that these barriers will be knocked down. ROS-based nanomaterials will break the shackle of lung cancer treatment, improve the prognosis of lung cancer and enhance the therapeutic effect of lung cancer.
